# Effects of deep brain stimulation of the subthalamic nucleus on inhibitory and executive control over prepotent responses in Parkinson's disease

**DOI:** 10.3389/fnsys.2013.00118

**Published:** 2013-12-25

**Authors:** Marjan Jahanshahi

**Affiliations:** Cognitive Motor Neuroscience Group and Unit of Functional Neurosurgery, Sobell Department of Motor Neuroscience and Movement Disorders, UCL Institute of Neurology, The National Hospital for Neurology and NeurosurgeryLondon, UK

**Keywords:** subthalamic nucleus, Parkinson's disease, deep brain stimulation, inhibition, executive control, prepotent responses

## Abstract

Inhibition of inappropriate, habitual or prepotent responses is an essential component of executive control and a cornerstone of self-control. Via the hyperdirect pathway, the subthalamic nucleus (STN) receives inputs from frontal areas involved in inhibition and executive control. Evidence is reviewed from our own work and the literature suggesting that in Parkinson's disease (PD), deep brain stimulation (DBS) of the STN has an impact on executive control during attention-demanding tasks or in situations of conflict when habitual or prepotent responses have to be inhibited. These results support a role for the STN in an inter-related set of processes: switching from automatic to controlled processing, inhibitory and executive control, adjusting response thresholds and influencing speed-accuracy trade-offs. Such STN DBS-induced deficits in inhibitory and executive control may contribute to some of the psychiatric problems experienced by a proportion of operated cases after STN DBS surgery in PD. However, as no direct evidence for such a link is currently available, there is a need to provide direct evidence for such a link between STN DBS-induced deficits in inhibitory and executive control and post-surgical psychiatric complications experienced by operated patients.

## Introduction

Parkinson's disease (PD) is the most typical basal ganglia disorder. In addition to the core motor symptoms of tremor, rigidity and bradykinesia and akinesia, patients experience a host of non-motor symptoms which include cognitive impairment and psychiatric disorders particularly depression, anxiety, apathy, hallucinations, and delusions. In relation to cognition, executive dysfunction can be present from the early stages of the illness and this and other forms of mild cognitive impairment can evolve into dementia in the later phases in a proportion of cases (Emre et al., 2007; Litvan et al., 2011, 2012; Dirnberger and Jahanshahi, 2013).

There is now evidence from randomized controlled studies that surgical treatment of PD with deep brain stimulation (DBS) of the subthalamic nucleus (STN) is effective in controlling the motor symptoms of the disease and improving the quality of life of the patients (e.g., Deuschl et al., 2006; Weaver et al., 2009, 2012; Follett et al., 2010; Williams et al., 2010). Also, a number of controlled studies have established that STN DBS does not produce any major deficits in global aspects of cognition in PD (e.g., Smeding et al., 2006; Witt et al., 2008; Weaver et al., 2009; Follett et al., 2010; Williams et al., 2010, 2011). Furthermore, the impact of STN DBS on cognition has been examined in a number of studies which have followed up patients for 5 (Schüpbach et al., 2005) 8 (Fasano et al. (2010), or 10 (Castrioto et al., 2011) years and the rates of dementia reported across these studies range from the 5–6 to 17–22%. These rates are no higher than those found as part of the natural history and progression of PD in longitudinal studies of cognition (Hughes et al., 2000; Aarsland et al., 2003; Hely et al., 2008), and suggest that STN DBS does not alter the risk of cognitive decline. The effect of STN DBS on more specific aspects of cognition was examined by Parsons et al. (2006) in a meta-analysis of 28 studies published between 1999 and 2006 based on 612 patients. The deterioration of verbal fluency with STN DBS was the most consistently reported change which had the highest effect size. There was also a small but still significant effect on verbal functions and executive functions.

Against this background, that STN DBS significantly improves the motor symptoms of PD and besides a deterioration of verbal fluency, STN DBS has no major negative impact on global cognitive function and is not associated with increased risk of cognitive decline, there is evidence that a specific aspect of executive function, executive control of action is impaired with STN DBS in PD, which is the focus of the rest of this review. To highlight these STN DBS induced deficits in executive control of action, the main part of the review reports the results of studies which have examined STN DBS effects on a range of tests including the Stroop, random number generation (RNG), stop signal task, go no go reaction times, and tasks involving decision-making under conflict. Where available, relevant imaging and electrophysiological recording studies are also discussed.

## Executive control over prepotent responses

Executive control is considered to be achieved by the frontal cortex in non-routine and demanding situations. Norman and Shallice (1986) defined situations that require executive control as those that involve planning or decision making, situations where responses are not well-rehearsed or contain novel sequences of actions or are dangerous or technically difficult, those that involve error correction or troubleshooting, and finally situations that require resisting temptation or overcoming of a strong habitual response. The latter situation requires inhibition of strong habitual responses to allow engagement in alternative behavior more suited to the context. Such strong habitual responses are prepotent, in that they are likely to be executed fast and automatically, without much attention or thought. Isoda and Hikosaka (2011) distinguished three mechanisms for development of prepotent responses. The first is an innate mechanism whereby a salient stimulus naturally and instinctively draws a response, such as the orienting response to a flashing light. The second, motivational prepotency, is elicited by a highly valued and immediately available reward as in the delayed discounting task or a food deprived dieter reaching and eating a doughnut when faced with a plate full of them. The third, habitual prepotency, is developed through repetition and practice; for example a driver stopping the car when the traffic light turns red.

A key feature of habitual prepotent responses is that they are executed fast (Schneider and Chein, 2003), presumably because they reach the threshold for execution before alternative responses. Thus, according to evidence accumulation models, the response threshold reflects the amount of information that needs to be accumulated before a response is made (e.g., Ratcliff, 1978). According to these models the speed and accuracy of responses are controlled by a change in the distance between baseline and response threshold levels. If the distance is short, the threshold will be reached quickly, but noisy inputs and incorrect activations are likely to reach threshold first, resulting in fast but error-prone responses. In contrast, if the distance is large, the threshold will be reached more slowly, with a smaller probability of incorrect activations reaching threshold first, such that responses will be made slowly but accurately (Bogacz et al., 2010). Control of such speed accuracy trade-offs (SAT) has been attributed to changes in baseline activity in cortical areas (pre-supplementary motor area—pre-SMA or dorsolateral prefrontal cortex), striatum or the STN or strengthening synaptic cortico-striatal connections [for review see Bogacz et al. (2010)]. There is some support from fMRI studies showing increased activation of the pre-SMA, striatum and STN with changes in response caution and SAT under different experimental conditions (e.g., Forstmann et al., 2008; Mansfield et al., 2011). According to Isoda and Hikosaka (2011), inhibitory control over habitual prepotent responses may simply delay or postpone it, to allow time for alternative more controlled responses to reach threshold.

Thus, an important aspect of executive control is inhibitory control, which encompasses the ability (i) not to react automatically to external stimuli, (ii) to exert control over internal impulses, and (iii) to prevent automatic performance of habitual responses in situations where more controlled processing is required. Impulsive individuals tend to act fast without reflection or foresight. However, impulsivity is multifactorial and various forms of impulsivity have been described including reflection impulsivity (act fast without taking time to reflect), impulsive action (inability to control prepotent responses as reflected by premature responses in go no go RT tasks and failure of motor inhibition in stop signal tasks) and choice impulsivity (failure of delayed gratification); which, respectively, operate at the preparation, execution and outcome stages of behavioral control (Evenden, 1999). The neural and neurochemical bases of these different forms of impulsivity have been recently reviewed (see Dalley et al., 2011; Dalley and Roiser, 2012).

Such inhibitory control over internal impulses or responses externally triggered by external stimuli or prepotent habitual responses is a cornerstone of self-control and essential for adaptive decision-making and appropriate social interaction. As outlined in the supervisory attentional system of Norman and Shallice (1986), inhibitory control over behavior is volitional and hence resource and attention-demanding. Inhibitory control over behavior can be reactive or proactive, operate globally or be selective, with these proposed to differentially engage the hyperdirect and indirect fronto-striatal pathways (Aron, 2011). Reactive inhibition is reflected for instance in the ability to stop oneself from continuing to cross the road if a fast car approaches and represents adaptive modification of behavior triggered by a sudden and unexpected stimulus. Proactive inhibition involves responding with restraint to meet goals and objectives. In the above example, proactive inhibition or action restraint would be the slowing down of one's walking pace when approaching the busy road. In daily life, proactive inhibition often concerns the preparedness to act with restraint in face of temptation or situations that challenge self-control such as drinking or smoking or eating sweets. Proactive inhibition is considered essential for self-control and most often goes awry in psychiatric disorders (Jaffard et al., 2008; Aron, 2011). In real-life situations, inhibitory control is often a key process in conflict resolution. The necessity to decide between equally salient or valued or incompatible options can induce a conflict. When faced with such conflict between available options, inhibitory control is imposed on responding, to prevent hasty decisions and premature responses until an optimal decision is arrived at (Frank, 2006). These inter-related inhibitory processes, reactive and proactive inhibition and conflict resolution, are essential for executive control and to ensure adaptive behavior (Frank, 2006; Verbruggen and Logan, 2009; Aron, 2011).

A factor analysis of different behavioral measures of impulsivity and risk taking has revealed two main factors. The first related to “impulsive action” and measures of inhibition of prepotent responses on go no go or stop signal tasks. The second factor corresponded to the “impulsive choice/decision” and measures of risk taking and delay discounting (Reynolds et al., 2006). In the same way that impulsivity is multi-faceted, inhibition is not a unitary concept and also has been shown to have different components. In their factor analytic study of nine different measures of inhibition on a sample of 220 students, Friedman and Miyake (2004) identified three factors which they labeled “inhibition of prepotent responses,” as measured by tasks such as the Stroop or stop signal RT task, “resistance to distractor interference” with tasks such as the Eriksen flanker task, and finally protection from “proactive interference” which measures resistance to memory intrusions from previously learned information with loadings from memory tasks such as the Brown-Peterson. Subsequently, the inhibition of prepotent responses and the resistance to distractor interference factors were shown to be related (*r* = 0.67) and were combined into a single factor. One aspect of RNG, suppression of habitual counting was found to be related to response-distractor inhibition. Thus, both inhibition and impulsivity are multi-faceted and here we are dealing with action impulsivity and inhibition of prepotent responses.

## The hyperdirect, direct and indirect pathways

**Figure 1:**
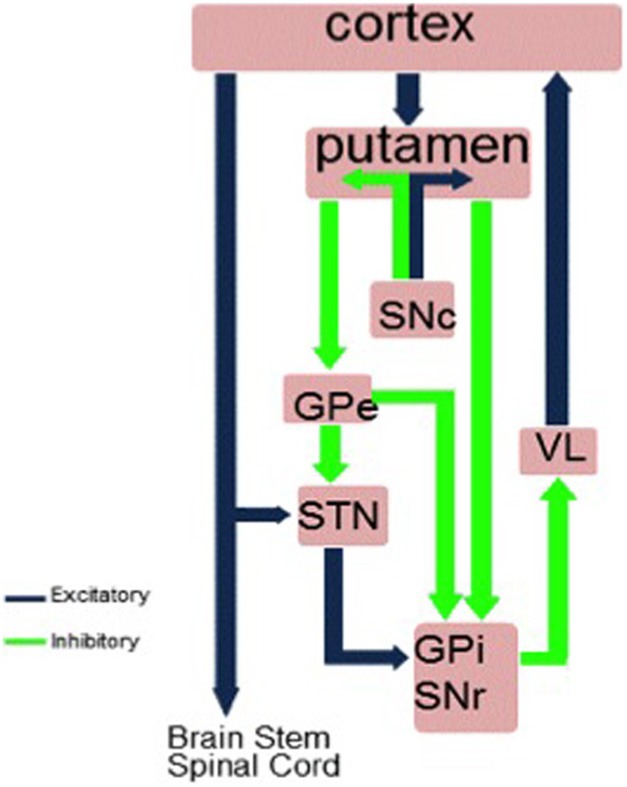
**The hyperdirect, direct and indirect pathways between the striatum and cortex.** GPi, internal segment of globus pallidus; GPe, external segment of globus pallidus; STN, subthalamic nucleus; SNc, substantia nigra pars compacta; SNr, substantia nigra pars reticulate; VL, ventrolateral thalamus.

The connectivity between the cortex and the basal ganglia occurs via three pathways: the hyperdirect, direct and indirect pathways (see Figure 1). These pathways have been considered to constitute an ideal system for response selection under competition or conflict. In situations of conflict, the hyperdirect pathway via the STN is proposed to increase the response threshold to prevent premature responses and to allow time for information accumulation/reflection and selection of the appropriate response, the indirect pathway via the STN inhibiting inappropriate responses to allow selection of the appropriate response through the direct pathway (e.g., Chevalier and Deniau, 1990; Mink and Thach, 1993; Redgrave et al., 1999; Frank, 2006; Frank et al., 2007).

In exerting inhibitory control over prepotent or habitual responses, the priority is to stop the prepotent response from being executed. The hyperdirect route from the cortex to STN is the shortest and fastest route for influencing the tonic inhibition of the basal ganglia output pathways over the cortex and achieving inhibition of action. The STN receives input from many frontal areas including the motor cortex, pre-SMA, caudal and dorsal premotor cortex, dorsolateral prefrontal cortex (DLPFC), and anterior cingulate cortex (ACC) and inferior frontal cortex (IFC) (Afsharpour, 1985; Parent and Hazrati, 1995; Nambu et al., 1997). Some of these areas such as the pre-SMA, IFC, ACC, and DLPFC are known to be involved in inhibitory control from investigations of the effects of accidental focal lesions in man (Devinsky et al., 1995; Aron et al., 2003; Dimitrov et al., 2003; Rieger et al., 2003; Sumner et al., 2007; Gläscher et al., 2012). This means that the STN is well-placed for a role in executive control through inhibition. Furthermore, recent imaging and tractography has revealed STN connectivity with the pre-SMA and IFC in man (Aron et al., 2007). Recent evidence from anterograde tracing studies in macaque monkeys suggests a topographically organized prefrontal-STN hyperdirect pathway, with limbic areas projecting to the medial tip of the STN, straddling its border and extending into the lateral hypothalamus and associative areas projecting to the medial half and motor areas to the lateral half; with limbic projections terminating rostrally and motor projections more caudally. A high degree of convergence existed between projections from functionally diverse cortical areas, which was considered to allow both functional specificity and integration (Haynes and Haber, 2013). Similar parcellation of the STN into three distinct zones was achieved *in vivo* and non-invasively in a study of brain connectivity profiles with diffusion weighted imaging which showed distinct limbic, associative and motor regions in the anterior, middle and posterior sections of the STN (Lambert et al., 2012).

Ablation of D2 receptor expressing striatal neurons in mice resulted in motor hyperactivity and examination of neuronal activity in the globus pallidus (GP) and substantia nigra pars reticulata (SNr) demonstrated that this ablation induced dramatic changes in the cortically evoked triphasic response of early excitation, inhibition and late excitation in the GP and SNr. It was concluded that the phasic late excitation in the SNr through the striatopallidal indirect pathway plays a key role in stopping movement and preventing motor hyperactivity (Sano et al., 2013).

Of interest is a recent study by Cui et al. (2013) which developed and used a novel *in vivo* photometry method to measure activity of spiny projection neurons in the direct and indirect pathways. Contrary to the classical model, of pro-kinetic “go” activity in the direct and anti-kinetic “no go” activity in the indirect pathway, during free movements in genetically engineered mice, there was co-activation of striatal neurons in both the direct and indirect pathways and spiny projection neurons in both pathways were quiet during inactive states. In an editorial on the Cui et al. paper, it was proposed that instead of issuing simple, generalized go or no go/stop commands, the co-activation of the direct and indirect pathways may be signaling “what to do” and “what not to do” and hence making recommendations about specific movements and their likely outcomes, which would enable selection of the optimal course of action (Surmeier, 2013).

## Models and predictions

Two models are relevant to a putative role for the STN in inhibitory and executive control. The first model proposed by Michael Frank (2006), Frank et al. (2007) considers the normal function of the STN to be to issue a “no go” signal to raise response thresholds when decision-making in situations of conflict to prevent premature and impulsive responding and to allow time for further information accumulation and reflection before a decision is made and a response is selected and executed. According to this model, alteration of STN activity as with STN DBS in PD should interfere with this normal function of the STN in raising the response threshold in situations of conflict and therefore be associated with impulsive responding.

A critical executive process is the ability to switch between automatic/habitual and controlled/goal-directed processing in a timely and efficient manner (Shiffrin and Schneider, 1984). Automatic habitual processing is employed when executing well-learned behaviors that require little attention. In contrast, when engaging in an attentionally-demanding behavior, such as when new learning is involved or when deliberately trying to override a well-learned habitual behavior, goal-directed controlled processing is necessary. It has been proposed that a critical function of the STN is switching between automatic and controlled strategies and that the STN receives a switching signal from regions of the prefrontal cortex, including the pre-SMA (Isoda and Hikosaka, 2008). Single cell recordings in primates during occulomotor tasks requiring such behavioral switching have revealed switch selective neurons in both the pre-SMA and the STN. Importantly, when a controlled response was required by the context, neurons in the pre-SMA fired *before* those in the STN, which in turn fired *before* response execution. Thus, recordings of neuronal activity in primates suggest that the STN implements a switch signal from the pre-SMA which enables a shift from habitual to controlled processing (Isoda and Hikosaka, 2008). Consequently, high frequency stimulation of the STN during STN DBS in PD may interfere with the ability to switch between automatic and controlled processing when a situation/task demands it.

## STN DBS in PD is associated with deficits in inhibitory or executive control over prepotent responses

In experimental animals such as the rat, lesions of the STN result in increased premature responses in reaction time tasks (Baunez et al., 1995), impulsivity and an inability to inhibit operant responses (Wiener et al., 2008), and a generalized impairment of stopping on a modified stop signal reaction time task (Eagle et al., 2008). In man, accidental lesions of the STN have resulted in hemiballism, hyperphagia, hypersexuality, loggoreha, eurphoria and impulsivity, symptoms indicative of motor and behavioral disinhibition (Trillet et al., 1995; Absher et al., 2000; Park et al., 2011).

While PD is primarily a disorder of response initiation characterized by akinesia or poverty of spontaneous and automatic actions such as blinking, gesturing, facial expression; and bradykinesia or slowness of movement initiation and execution; some of the other symptoms of PD such as freezing of gait or medication-induced dyskinesias represent excessive inhibition or disinhibition of movement, respectively. Patients with PD have been shown to have deficits in inhibitory control on tasks requiring inhibition of prepotent motor response such as go no go RT (Cooper et al., 1994) or stop signal RT (Gauggel et al., 2004; Obeso et al., 2011a,b), as well as inhibition on cognitive tasks requiring inhibition of prepotent or habitual responses such as the Stroop, RNG or the Hayling sentence completion task (Obeso et al., 2011a).

There is now an increasing body of evidence suggesting that treatment of PD with STN DBS is associated with deficits in inhibitory and executive control. This literature largely based on an STN DBS on vs. off methodology sometimes combined with imaging or involving recording of local field potentials (LFPs) from electrodes surgically implanted in the STN or intraoperative recording of single neuronal activity from the STN has relied on the use of a variety of tasks and will be reviewed below according to the specific tasks employed.

### The stroop interference task

**Figure 2:**
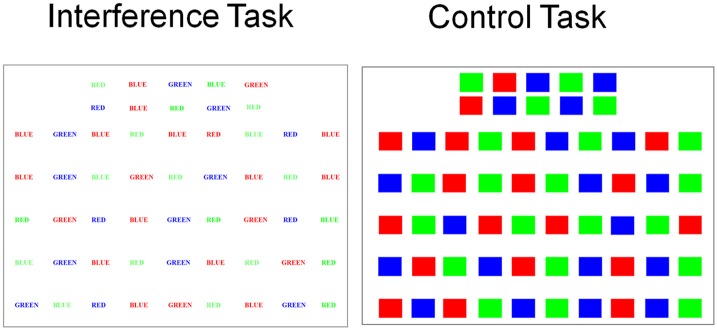
**Examples of stimuli used for the Stroop interference (color words red, blue, and green printed in incongruent ink) and control (rectangles printed in red, blue, or green) tasks**.

The Stroop interference task (Stroop, 1935) is a classic example of a task that involves response selection under conflict and requires inhibitory control over habitual prepotent responses in order to select an alternative response. Reading words is a habitual prepotent response built up through years of exposure to printed words. In the Stroop interference task (Figure 2), the color words, red, blue and green are presented in incongruent ink. For example the word red is printed in blue ink. The participants' instruction is to name the color of ink the word is printed in. To do this, the participant has to suppress the more habitual and prepotent response of reading the word (red), in order to select the alternative response and name the color of ink it is printed in (blue). As a result, participants take longer to complete and make more errors on the Stroop Interference task than on a control task when they name the color of ink of colored rectangles printed in red, blue or green.

Jahanshahi et al. (2000a) used a DBS on vs. off methodology to assess seven patients with PD who had had STN DBS and 6 with GPi DBS. Patients were assessed after overnight withdrawal of dopaminergic medication in the off state. While PD patients were significantly faster on the Stroop control task with STN DBS on than off, with STN DBS on, they made significantly more errors on the Stroop interference task, compared to when the stimulators were off. These effects were not significant for the patients with GPi DBS. This was the first experimental demonstration that STN DBS induced deficits in inhibitory control and an inability to suppress habitual prepotent responses and to engage in response selection under conflict in PD. These findings were subsequently replicated by others (Witt et al., 2004).

With PET, Schroeder et al. (2002) assessed the neural substrates of this DBS STN-induced deficit in inhibitory control on the Stroop interference task. Seven patients with PD performed the Stroop task or a control task naming animals, also printed in colored ink. With STN stimulation on the Stroop interference effect, the difference between completion of the Stroop interference and control tasks, was significantly larger than with DBS off. This greater interference effect and inability to inhibit the prepotent response with STN DBS was associated with increased activation of the left angular gyrus an area related to processing of words which was activated more with STN stimulation reflecting the increased inability to suppress processing of the words probably related to the reduced activation of the ventral striatum and anterior cingulate observed with STN stimulation compared to DBS off. This suggests that the inability to inhibit prepotent word reading responses and to engage in response selection under conflict on the Stroop interference task is mediated by reduced activation of the limbic circuit induced by STN stimulation.

More recently, Brittain et al. (2012) recorded LFPs from the electrodes implanted in the STN of 12 medicated PD patients in the few days post-operatively when this is possible before the electrodes are connected to the impulse generating device. They used a computerized version of the Stroop interference task with color words presented in incongruent ink and a control task in which color words were presented in congruent ink. As expected, during incongruent trials, response times were longer (980 ms) and error rates higher (6.5%) due to the Stroop effect than on the congruent trials (807 ms error rate 1.3%). They found stimulus driven beta desynhronization (15–35 Hz) that lasted throughout the verbal response—consistent with the idea that beta synchrony decreases to allow motor output to occur. On the incongruent trials there was a rebound in beta desynchrony between −300 and 100 ms prior to the response which was not present for the congruent trials. They then looked at response locked change in beta power for correct and incorrect incongruent trials relative to baseline. On correct incongruent trials when the prepotent response was successfully inhibited, a beta resynchronization was seen before the response. During incorrect incongruent trials when the patient failed to inhibit the prepotent response, the beta resynchronization occurred after the response onset. On correct trials, the beta power reshyncronization occurred at a mean −138 ms before the response, whereas for the incorrect response, this occurred 132 ms after the response. It was suggested that this beta resynchronization or rebound during incongruent trials is an inhibitory signal via the hyperdirect pathway which pauses the motor system and delays the prepotent response until the conflict can be resolved and a correct response is selected and produced.

### Random number generation

Random number generation (RNG) is procedurally simple. Participants are instructed to say the numbers 1–9 in a random fashion, as if picking them out of a hat, in synchrony with a pacing stimulus for 100 trials. RNG involves a number of executive processes. As there are nine possible responses to select from on each trial, RNG involves response selection under conflict. In addition, to engage in strategic response selection in a random fashion, participants have to suppress habitual counting in series (e.g., 123 or 987, measured as count score 1- CS1) which is a prepotent habitual response, developed through years of experience with numbers. Selection and switching generation strategies, monitoring of the output and synchronizing responses with the pacer are other processes involved in the task. As a result, RNG is an attention-demanding task that interferes with performance of other attention-demanding tasks and in turn is subject to interference when performed concurrently with other such tasks under dual-task conditions (Baddeley, 1966; Robertson et al., 1996; Brown et al., 1998). In fact, during such dual task conditions or when paced RNG is performed at faster rates which also increases attentional demands of the task, there is significant increase in habitual counting (CS1) during RNG, indicating that participants are less able to suppress habitual counting and engage in strategic response selection (Brown et al., 1998; Jahanshahi et al., 1998, 2000b, 2006; Dirnberger et al., 2005). With imaging, it has been shown that in healthy young individuals, performance of paced RNG at the fastest rates, requiring a response once every 1 or 0.5 s, is associated with significant increase in habitual counting (CS1) and decrease in frontal activation, presumably because the response selection and synchronization demands of the task exceed capacity (Jahanshahi et al., 2000b). Patients with PD, who show differentially greater increase in habitual counting at faster rates of paced RNG relative to age-matched controls, unlike the controls fail to show task or rate-dependent modulation of frontal activation, a dysfunction related to group differences in GPi activation across tasks and rates (Dirnberger et al., 2005).

**Figure 3:**
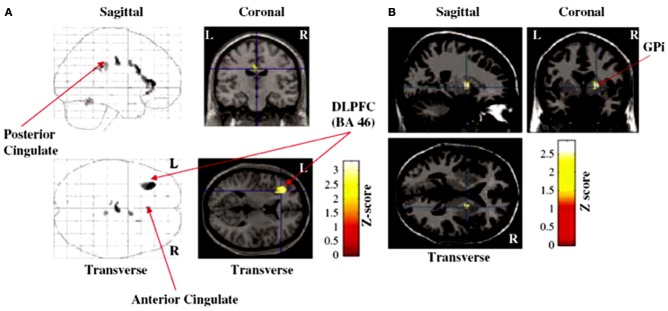
**Brain areas showing significant **(A)** decreased activation [dorsolateral prefrontal cortex (DLPFC), anterior cingulate cortex, posterior cingulate cortex] or **(B)** increased activation right internal segment of the globus pallidus (GPi) with subthalamic stimulation on compared to deep brain stimulation off during performance of the paced random number generation task by patients with Parkinson's disease in the study of Thobois et al. (2007)**.

**Figure 4:**
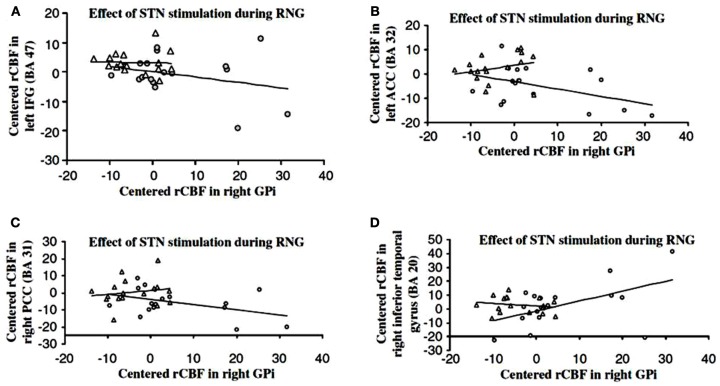
**Psychophysiological interactions showing negative coupling between the right internal segment of the globus pallidus (GPi) and the **(A)** inferior frontal cortex (IFC), **(B)** anterior cingulate cortex (ACC), **(C)** posterior cingulate cortex (PCC) and positive coupling between the right GPi, and the **(D)** left temporal cortex with the subthalamic nucleus deep brain stimulation (DBS) on (longer lines) (shorter lines are DBS off) during performance of the paced random number generation task in patients with Parkinson's disease in the study of Thobois et al. (2007)**.

Thobois et al. (2007) used PET to investigate the effect of STN DBS in PD on patterns of brain activation during fast-paced RNG or a control counting task (counting in series from 1 to 9) both paced by a 1 Hz tone. While STN DBS significantly improved the motor symptoms of PD, compared to DBS off, patients engaged in significantly higher habitual counting (CS1) with STN stimulation. STN DBS did not influence synchronization with the pacing stimulus as measured by the total time taken to complete the RNG task. STN stimulation was associated with significant increase in activation of the right GPi, and significant decreased activation in the left DLPFC, the left posterior cingulate and anterior cingulate during fast-paced RNG (see Figure 3). Furthermore, the measure of habitual counting during RNG, CS1 was significantly and negatively correlated with activation in the left anterior cingulate (BA 32), left inferior frontal gyrus (BA 47) and the left posterior cingulate (BA 23), indicating that reduced activation in these areas was associated with increased habitual counting during RNG. Using the right GPi as the seed area, psychophysiological interactions showed that STN stimulation was associated with negative coupling between the GPi and the left inferior gyrus, the left anterior cingulate and the right posterior cingulate (see Figure 4). These results were the first demonstration that STN stimulation interfered with inhibitory control over habitual responses and strategic response selection under conflict by altering pallidal-frontal-cingulate coupling during performance of the fast-paced RNG.

More recently, Anzak et al. (2013) recorded LFPs bilaterally from the electrodes implanted in the STN in 7 PD patients in the immediate post-operative phase while the patients performed 6 trials of either a paced (0.5 Hz) RNG or a control counting task. Performance of the paced RNG was associated with a significant increase in gamma band power in the 45–60 Hz range relative to the control counting task. Furthermore, STN LFP increases in the gamma band during RNG were significantly and positively correlated with the number of repeated pairs (a measure of controlled processing during RNG which participants engage in at slower rates of paced RNG when there is time for controlled processing and volitional repetition of the same number across successive trials) and negatively correlated with CS1 (the measure of habitual counting and automatic processing during RNG), suggesting that the higher gamma power change may represent a switch from automatic to controlled processing during the RNG task. These results directly relate measures of switching from automatic to controlled processing during the RNG task (indexed by the CS1 and repeated pairs measures, respectively) to modulation of activity in the STN itself.

### Stop signal RT task

**Figure 5:**
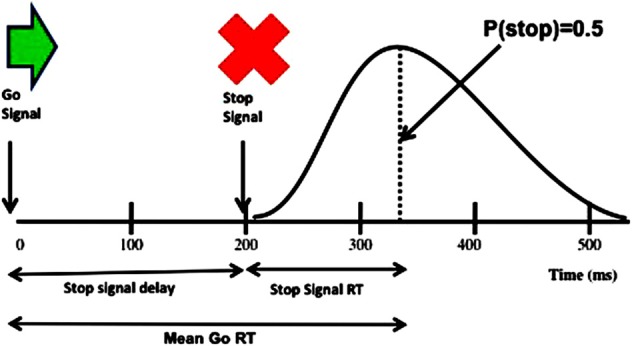
**Schematic representation of the stimuli on the stop signal task (upper part) and also showing the reaction time (RT) distribution and how the stop signal reaction time (SSRT) is derived as the difference between mean Go RTs and the average stop signal delay (SSD) at the point when responses are successfully inhibited on 50% of the trials (lower part)**.

Another task that requires inhibition of prepotent responses is the stop signal reaction time (RT) task (Logan and Cowan, 1984), in which a stop signal presented at variable stop signal delays after a go signal, instructs participants to inhibit the response prepared following the go signal which may be close to execution and hence prepotent (Figure 5). This task has been widely used to measure reactive inhibition, through estimation of the stop signal reaction time (SSRT), on the basis of the “horse race” model which proposes that the outcome of the race between the go and the stop process determines whether the participant successfully stops or fails to stop and responds on the stop trials. Imaging studies in healthy participants have shown that successful motor inhibition on the stop signal task is associated with increased activation of frontal areas such as the pre-supplementary motor area (pre-SMA), IFC and the anterior cingulate as well as the striatum and the STN (Rubia et al., 2003; Aron and Poldrack, 2006; Li et al., 2006, 2008; Aron et al., 2007; Zandbelt and Vink, 2010). Furthermore, in an fMRI study using a *conditional* version of the stop signal task, significant activation of a right-hemispheric “braking” network of STN, IFC, and pre-SMA was described, in association with both reactive inhibition in response to a stop signal on “critical” trials and conflict-induced slowing on “non-critical” trials when the stop signal was presented but had to be ignored (Aron et al., 2007).

Several studies have examined the effect of STN DBS in PD on performance of the standard version of the stop signal task and the SSRT, the measure of inhibition usually derived by applying the race horse model and using a staircase tracking procedure and integration method by subtracting the average stop signal delay (interval between go and stop signals) from the mean go RTs. van den Wildenberg et al. (2006), Swann et al. (2011), Mirabella et al. (2012) reported that SSRTs were significantly shorter with stimulation than with STN DBS off, suggesting that STN DBS *improves* inhibitory control on the stop signal task. In contrast, Ray et al. (2009) found that when PD patients were equated with healthy controls for baseline SSRTs, STN DBS was associated with longer SSRTs relative to DBS off. While reactive inhibition (e.g., stopping car when traffic light turns red) is required in some real life situations, proactive inhibition, the ability to act with restraint (e.g., not to eat a piece of cake when dieting) and to inhibit impulsive responding in situations of conflict to allow time for reflection are also relevant to daily life. Using the conditional version of the stop signal task, Obeso et al. (2013) examined the impact of STN DBS in PD on proactive inhibition and conflict-induced slowing as well as the SSRT measure of reactive inhibition. They found that SSRTs were significantly prolonged with STN stimulation relative to DBS off, whereas relative to healthy controls proactive action restraint was significantly lower with DBS off but not DBS on. While the mean measure of conflict-induced slowing was not altered by STN DBS, stimulation produced a significant differential effect on the slowest and fastest RTs on conflict trials, further prolonging the slowest RTs on the conflict trials relative to DBS off and to controls. These results indicate that STN DBS produces differential effects on reactive and proactive inhibition and on conflict resolution. These differential effects of STN stimulation on the various measures of inhibitory and executive control may be mediated through the hyperdirect, indirect and direct pathways, consistent with imaging evidence that while the hyperdirect pathway via the STN is crucial for temporary “hold your horses” braking or global reactive inhibition, proactive and selective inhibitory control may be mediated via the striatum (Jahfari et al., 2010, 2011; Zandbelt and Vink, 2010; Aron, 2011).

A number of factors may contribute to these inconsistent results of STN DBS on SSRTs across studies. First, the specific type of stop signal RT task used, the nature of stimuli and responses, crucial timing features and the proportion of go and stop trials that would have influenced the prepotency of the response and hence the difficulty of stopping, varied across studies. Second, the results of Ray et al. (2009) indicate that baseline SSRTs relative to controls may be important in determining the direction of the effects of STN stimulation, with those having similar baseline (DBS off) SSRTs as controls showing prolongation of SSRT with stimulation, whereas STN stimulation speeded up SSRTs for patients with slower baseline SSRTs relative to controls. Third, the precise effects of STN DBS on SSRT are likely to depend on the exact location of the active contacts used for stimulation. It has been shown that active contacts which are in the dorsal vs. ventral parts of the STN produce distinct effects on inhibitory processing on a go no go RT task (Hershey et al., 2010), with stimulation of contacts in the ventral STN inducing a greater inhibitory deficit. However, with the stop signal RT task, Greenhouse et al. (2011) did not find any differences in SSRT with stimulation of the most ventral vs. the most dorsal contacts in PD patients. Future studies relating the exact location of contacts in the STN to effects on SSRT can clarify this issue. Fourth, procedural variations such as whether DBS was unilateral (Ray et al., 2009) or bilateral (van den Wildenberg et al., 2006; Swann et al., 2011; Mirabella et al., 2012), whether stop signal task performance was unimanual (present study; Ray et al., 2009; Mirabella et al., 2012) or bimanual (van den Wildenberg et al., 2006; Swann et al., 2011), the type of movement performed (reaching, Mirabella et al., 2012 or manual keypress, van den Wildenberg et al., 2006; Ray et al., 2009; Obeso et al., 2013), whether patients were assessed on (van den Wildenberg et al., 2006; Ray et al., 2009; Swann et al., 2011; Obeso et al., 2013) or off (Mirabella et al., 2012) medication are important methodological differences across studies that would have influenced the results.

Event-related potentials from surface EEG have been recorded during performance of the stop signal with STN DBS on and off in one study of PD patients (Swann et al., 2011), while two other studies have recorded LFPs from electrodes implanted in the STN in PD patients during performance of the stop signal task (Ray et al., 2012; Alegre et al., 2013). Swann et al. (2011) examined stop signal RTs in 13 PD patients with bilateral DBS of the STN assessed on medication and 14 healthy controls and recorded 64 channels of scalp EEG during performance of the task. The only measure that was significantly altered by DBS of the STN was SSRT, which was significantly improved/faster with DBS on relative to DBS off. They found increased beta band power (considered to be “anti-kinetic”) around the time of stopping with STN DBS on relative to off stimulation over the right frontal cortex. Furthermore, increased beta band activity over the right frontal cortex was noted on successful compared to unsuccessful stop trials. It was concluded that STN DBS alters the fidelity of information transmission in the subthalamic-cortical pathways which influences inhibitory control over action.

**Figure 6:**
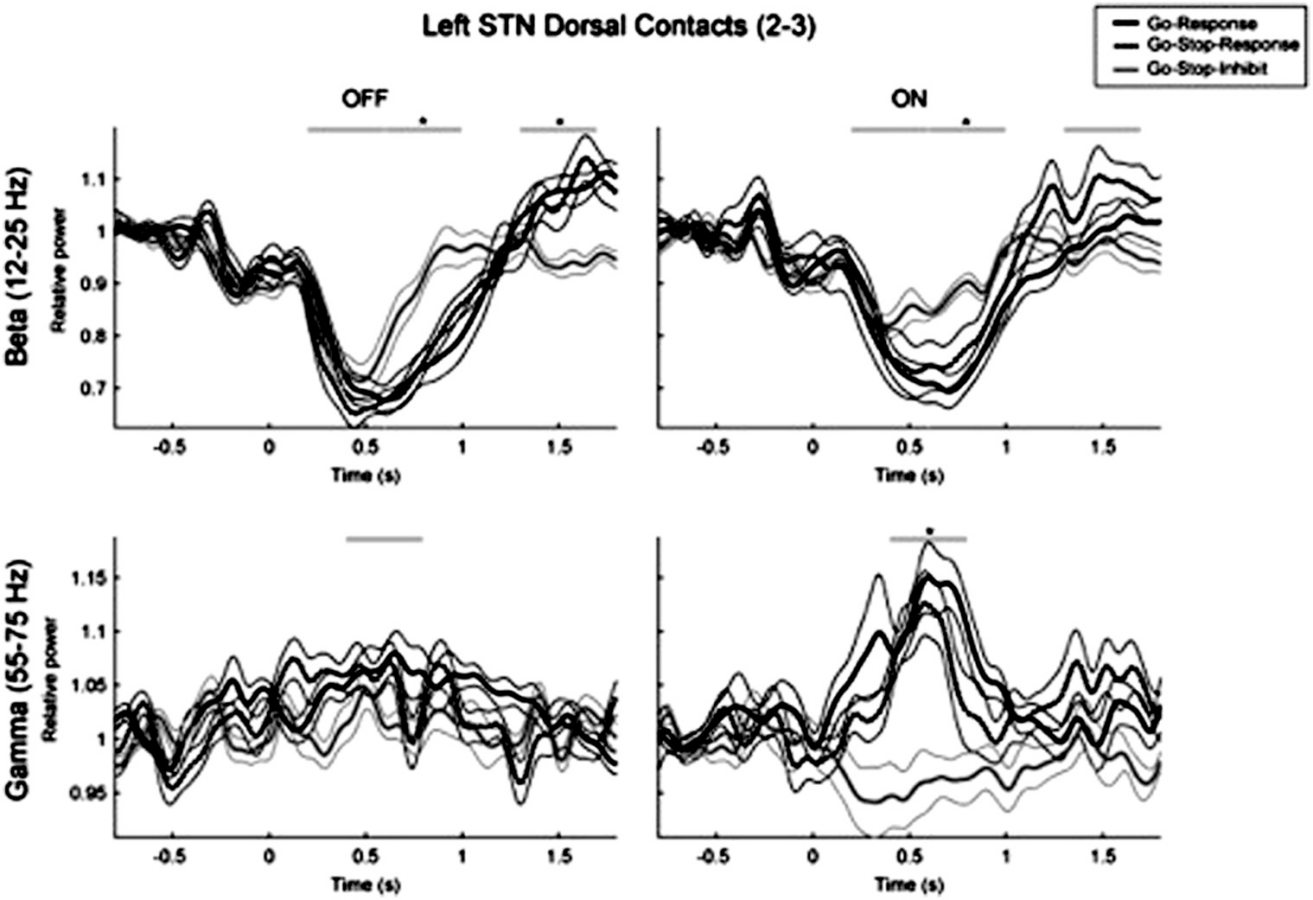
The changes in the gamma band activity in local field potentials recorded from the implanted electrodes in the subthalamic nucleus when patients with Parkinson's disease were assessed on and off levodopa medication during performance of the stop signal RT task. ^*^ denotes significant differences between the different types of trial.

LFPs from the STN during a stop signal task with an auditory stop signal presented on 25% of trials were recorded by Ray et al. (2012) in 9 PD patients assessed on medication. Presentation of the stop signal was associated with increase in beta activity or beta event-related synchronization (ERS), but beta ERS after the stop signal was not different for failed vs. successful inhibition trials. However, once the influence of stop signal delays was removed, the time point at which beta synchrony increased during successfully inhibited trials correlated with SSRT suggesting that those with quicker onset of beta synchrony following the stop signal had shorter SSRTs. Gamma ERS was noted following go signals and was also evoked by stop signals. Gamma ERS was highest for failed stop trials and less for successfully inhibited trials, similar to the results of Alegre et al. (2013) discussed next. In 10 PD patients Alegre et al. (2013) recorded LFPs from the STN during performance of a stop signal task (50% stop trials) both on and off dopaminergic medication. Response preparation was associated with decrease in beta power (12–30 Hz) and cortico-subthalamic coherence in beta band, which was smaller and shorter when the response was successfully inhibited. In the theta band there was an increase in frontal-cortico-subthalamic coherence related to the presence of the stop signal which was higher when the response inhibition was unsuccessful, perhaps reflecting the conflict between performance and inhibition of an action. A differential pattern of gamma activity was seen on medication (see Figure 6). Performance of the response was associated with a significant increase in power (55–75 Hz) and cortico-subthalamic coherence, whereas successful inhibition of the response was associated with bilateral decrease in subthalamic power and cortico-subthalamic coherence. Importantly, the inhibition related decrease in gamma activity was absent in the four patients with dopamine agonist related impulse control disorders (ICDs). These results were interpreted as supporting involvement of STN in response inhibition in the stop signal task and suggesting that this may be mediated by a reduction of gamma oscillations in the cortico-subthalamic connection which may reflect the suppression of the intention to move.

On a modified version of the stop signal task, following excitotoxic lesions of the STN, rats showed a failure to activate the stop process, which resulted in a larger number of errors. In contrast, the SSRT, the main measure of inhibition on this task, was not affected by such STN lesions (Eagle et al., 2008). STN lesions also speeded up responses on go trials and reduced the accuracy of stopping for all SSDs, suggesting a more generalized stopping impairment (Eagle et al., 2008). Of great interest is the recent study by Schmidt et al. (2013) who recorded neuronal activity from multiple basal ganglia structures in rats during a modified version of the stop signal task. Striatal neurons became active on presentation of go cues but not stop cues. STN neurons had low latency responses to stop cues, on both stop success and stop failure trials, suggesting that the STN provides fast signals to stop action, whether this is successful or not. SNr neurons responded to stop cues on trials on which the response was successfully inhibited. Based on these results and simulations, it was suggested that the results support the race model, particularly the interactive race model of the go and stop processes. The outcome of the race between the stop and go processes is determined by the relative timing of the distinct inputs to SNr neurons from the striatum and STN. If the GABAergic signal from the striatum arrives first and wins the race then SNr pauses firing and a response is made (success failure trial). In contrast, if the glutamatergic excitatory signal from the STN wins the race, then the SNr increases firing and the response is withheld (stop success trial). It was further proposed that the STN may be part of a broader “interrupt” system mediated by the centromedian and parafascicular thalamic nuclei and/or pendunclopontine nucleus that coordinates responses to salient cues across multiple timescales and pathways.

### Go no go RTs

While stop signal RTs measure volitional inhibition and cancellation of a prepared action, Go no go RTs are a measure of withholding a response or action restraint. Many variations of the Go no Go RT task exist. Essentially, across trials patients are instructed to respond to most stimuli presented but to withhold their response to a specific stimulus. For example, press a button when a green square (go stimulus) is presented, but do not respond when a red square appears (no go stimulus). The proportion of go to no go stimuli in a block determines, the degree of motor preparation, and withholding responses becomes more difficult when motor readiness is increased with a higher percent of go trials in a block.

A number of studies have examined the influence of STN DBS in PD on go no go RTs, using a variety of approaches: behavioral, imaging and recording of LFPs. Using go no go RT tasks with 83 or 50% go trials, Hershey et al. (2004) provided evidence that STN DBS in PD patients assessed off medication selectively interfered with action restraint during the blocks with a higher percent of go trials, when the response was more prepotent; as patients had significantly higher commission errors and lower discriminability index with DBS on only during these trials. These results suggest that STN stimulation interferes with action restraint under conditions of high demand on executive control. Subsequently, Kuhn et al. (2004) recorded LFPs from the STN in 8 PD patients performing a go no go RT task (20% no go trials). As expected, beta band activity decreased prior to movement on go trials and was followed by a late post-movement increase in beta power. In contrast, on no go trials, the beta power drop following presentation of the imperative signal was prematurely terminated and reversed into beta power increase. When go trials were subtracted from no go trials, the difference was evident as beta power increase. These results suggest that changes in LFP activity in the STN in the beta band may be important for determining whether movement is initiated or withheld.

In a second behavioral study with the go no go task (83% go), Hershey et al. (2010) assessed the effects of unilateral stimulation through electrodes contralateral to the worst side of the body to compare the effects of stimulation through contacts which were in the dorsal vs. ventral sections of the STN on performance of 10 PD patients tested off medication. As in their previous study, patients responded to all letter stimuli but withheld a response when the number 5 was presented on 17% of the 150 trials. The same stimulation parameters were used for all dorsal and ventral contacts. Compared to a no stimulation condition, both dorsal and ventral STN stimulation resulted in significant reduction of UPDRS scores and improvement of motor symptoms of PD. While the go RTs did not differ for ventral vs. dorsal stimulation the discriminability index, which is based on the proportion of hits minus the proportion of false alarms was significantly lower with DBS through the ventral than the dorsal contacts. Only ventral stimulation decreased hits and increased false alarms on the go no go task. These results were interpreted as indicating that the ventral part of the STN is involved in the balance between selection and inhibition of prepared responses.

**Figure 7:**
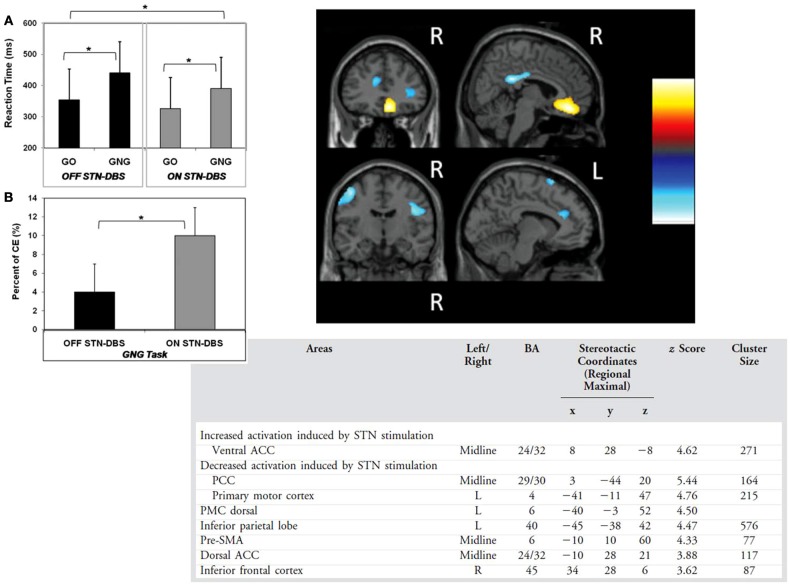
**(A)** The reaction times for the go no go (GNG) and simple reaction time (GO) tasks **(B)** commission errors (CE) in the go no go task with subthalamic nucleus deep brain stimulation on or off **(C)** areas showing decreased and increased activation with subthalamic nucleus stimulation across the two tasks in the study of Ballanger et al. (2009). ACC, anterior cingulate cotex; PCC, posterior cingulate cortex, R, right; L, left; ^*^ denotes significant differences (see text).

Using PET, Ballanger working with the Toronto group (Ballanger et al., 2009) examined the neural correlates of inhibitory control with STN DBS in PD to compare two different but not mutually exclusive frameworks of phasic “hold your horses” reactive inhibition in situations of conflict and more tonic “proactive inhibition” relating to situations of uncertainty, which make different predictions in terms of the brain areas involved and the time course of inhibition. According to the proactive inhibition model, a simple RT task which does not entail any conflict nevertheless engages proactive inhibitory control to withhold the response in advance of presentation of the go stimulus which signals release of inhibitory control over the response. They used an STN DBS on vs. off methodology to investigate STN modulation of go no go RTs (Go: white circle, No Go: white X, 40% no go) and simple RT (Go: white circle) in 7 PD patients tested off medication. As expected, STN DBS improved the motor symptoms of PD and speeded up RTs for the simple RT block as well as blocks with a mixture of Go and Go RTs. But at the same time STN stimulation increased errors of commission on the no go trials, indicating that patients were less able to inhibit the prepotent response. STN stimulation was associated with a significant increase in activation in subgenual ACC (BA24/32) and decreased activation in the medial posterior cingulate cortex (BA 29/30), pre-SMA (BA 6), dorsal ACC (BA 24/32) and left primary cortex (BA 4), inferior parietal lobe (BA 40), dorsal premotor cotex (BA6) and right ventral premotor cortex (BA 6) and IFC (BA 44) (see Figure 7). The main effect of Task and the Task x Stimulation interaction were not significant, suggesting the STN-induced changes in brain activation held for both the simple and go no go RTs, respectively, involving predominantly proactive and reactive inhibitory control. The number of commission errors were significantly associated with activation in the precuneus. The increased activation in the subgenual ACC, an area associated with impulsive behavior in bipolar disorder (e.g., Swann et al., 2003) was considered to reflect increased motivational drive induced by STN DBS in PD. It was concluded that the results supported STN stimulation having an effect on brain areas mediating both reactive (ACC, pre-SMA, premotor cortex, IFC), and proactive inhibition (posterior cingulate, precuneus, inferior parietal cortex) inhibitory processes.

In a subsequent behavioral study by some of the same group, Favre et al. (2013) formulated bradykinesia in PD in terms of a deficit in release of proactive inhibitory control. They used warned (150, 350, 550 ms warning-go signal interval) and unwarned simple RT tasks in a mixed block or a pure block of unwarned RTs. They reported that relative to controls, the PD patients were impaired in releasing proactive inhibition when this was internally driven in an unwarned simple RT condition which was considered responsible for their slowness in movement initiation. While dopaminergic medication generally improved RTs, medication status did not influence the internal control of the proactive inhibition, whereas DBS of the STN restored voluntary release of the proactive inhibition. The results of Favre et al. (2013) together with the findings of Obeso et al. (2013), suggest that STN DBS in PD also influences proactive inhibition. As proactive inhibitory control is more relevant to self-control in daily life, this may be more pertinent to understanding development of psychiatric problems such as emergence of de novo ICDs following STN surgery (Smeding et al., 2007; Halbig et al., 2009; Lim et al., 2009). However, paradoxically, both Favre et al. (2013) and Obeso et al. (2013) suggest that proactive inhibitory control is better with STN stimulation than with DBS off.

### Decision making and conflict resolution

Normally, when making decisions in situations of conflict, individuals take time to reflect and weigh up the available options and the desirability of their likely outcomes, which slows down their responses before a decision is made. To test the hypothesis outlined above (Frank, 2006; Frank et al., 2007) that in situations of conflict, the STN issues a global “no go” signal to temporarily brake responding and prevent impulsive action, to allow time for information accumulation and reflection before a choice is made, Frank et al. (2007), Cavanagh et al. (2011) have completed a number of studies on patients with PD with bilateral STN DBS. The same task was used which involved probabilistic decision-making. When faced with high conflict stimuli with equally high (e.g., 80 vs. 70%) probability of reward, elderly controls and unoperated PD patients on and off medication and the operated patients tested with DBS off slowed their response and had longer RTs relative to the low conflict condition with stimulus pairs with more discriminable reward values. In contrast, the patients with STN DBS on had faster RTs in the high than low conflict situations and so acted impulsively particularly when the stimuli were associated with a high probability of reward.

A later study from the same group using the same probabilistic decision making task, incorporated recording of scalp EEG with an STN DBS on vs. off methodology (*N* = 17) and intraoperative recording from a stimulating electrode inserted in the STN (*N* = 8). Cavanagh et al. (2011) showed that ordinarily the increase in theta band activity (4–8 Hz) over the medial prefrontal cortex is associated with raising the decision threshold for high conflict trials but not low conflict trials and that STN DBS in PD reversed this relationship such that increased theta band activity was associated with a decrease of the decision threshold on high conflict trials. It was proposed that the medial prefrontal cortex and the STN communicate in low frequency bands to represent decision conflict and that STN DBS interferes with the normal ability of the STN to react to decision conflict by modulating the decision threshold.

Further evidence for the Frank model and the STN role in decision-making under conflict has been provided by other groups. Fumagalli et al. (2011) recorded LFPS from the STN in 16 PD patients during processing of moral conflictual (e.g., “abortion is murder”), moral non-conflictual (“all men have a right to live”) and neutral (e.g., “a piano has black and white keys”). RTs were significantly longer for moral conflictual than moral non-conflictual statements. Relative to baseline, there was a significant increase in low frequency power in the 5–13 HZ band in all conditions, which was significantly higher for the moral conflictual sentences. Intraoperative microelectrode recordings of single unit activity from the STN during DBS surgery in 14 PD patients off medication performing a probabilistic decision making task showed increased spiking activity in the STN when the patients engaged in a decision. Importantly, the level of STN spiking activity increased with the level of decision conflict and seemed to be related to choice difficulty and accuracy rather than reward association (Zaghloul et al., 2012). Coulthard et al. (2012) employed a task involving learning and probabilistic decision-making under conflict and examined the effect of STN DBS in 11 PD patients. They found that while STN stimulation did not affect the learning phase, it influenced the information integration phase when participants needed to update their decision on the basis of previous pieces of information presented and with STN DBS on patients failed to slow down to revise their plan, reflecting impulsivity.

To date, only one study has directly related STN activity during decision-making to presence of ICDs in PD. Rosa et al. (2013) used an economic decision-making task with conflictual and non-conflictual trials and recorded LFPs from the STN in PD patients, 8 of whom had pathological gambling. Based on an index of risk, 3 types of behavioral strategy—risky, random and non-risky- were distinguished, that were, respectively, employed by 6 patients with pathological gambling, 5 patients (3 with pathological gambling and 2 without), and 6 patients without pathological gambling. The subgroup with pathological gambling who engaged a risky strategy had a significantly higher change in low frequency power in the STN when evaluating conflictual vs. non-conflictual stimulus pairs. Such a difference was not observed for the patients without pathological gambling who adopted a non-risky strategy. These results directly relate low frequency STN activity to adoption of risky strategies in patients with PD and pathological gambling. Similar to these findings from Rosa et al. (2013), Rodriguez-Oroz et al. (2011) also merged the two strands of research on impulsivity in PD, respectively, focused on ICDs and STN DBS induced inhibitory deficits. They recorded LFPs from the STN and reported that on medication, 10 PD patients who had ICDs before surgery had theta-alpha (4–10 Hz) activity generated 2–8 mm below the intercommissural line from the ventral contacts and cortico-subthalamic coherence in the 4–7.5 Hz range in scalp electrodes over the prefrontal cortex.

Another task that requires perceptual decision-making is the “moving dots” task, which consists of a cloud of dots, a proportion of which move coherently in one direction, whereas the rest move randomly. The participant has to decide whether the dots are moving to the right or left. The task has been shown to involve modulation of response thresholds under speed and accuracy instructions, with response caution being associated with increased activation of the pre-SMA and the putamen (Forstmann et al., 2008). The moving dots task was used by Green et al. (2013) to investigate the impact of STN DBS in PD on modulation of response thresholds and speed-accuracy trade-offs under speed vs. accuracy instructions with six levels of task difficulty achieved by altering the degree of coherence of the moving dots, with the low coherence conditions considered equivalent to situations of high conflict. They found that with STN stimulation patients were faster but less accurate compared to DBS off, and STN DBS altered the degree to which patients were able to adjust their decision thresholds as a function of task difficulty/conflict. Similar changes in RTs and accuracy using this task have been obtained in our laboratory (Pote et al., in preparation) on 12 PD patients with STN DBS. Furthermore, application of the drift diffusion model showed that stimulation of the STN was associated with lowering of response thresholds under speed instructions only, such that the patients had differentially faster RTs but were less accurate under speed instructions compared to DBS off. While non-decision time was higher with DBS off than on, STN DBS did not influence drift rate which for the PD patients was almost half that observed for the healthy controls. With somewhat different decision-making tasks, the results of the Cavanagh et al. (2011), Green et al. (2013), and Pote et al. (in preparation) concur that STN DBS in PD lowers response thresholds during decision-making tasks which accounts for the fast and errorful reactions of the patients with the stimulators switched on when faced with decision conflict, task difficulty or time pressure.

### Other tasks involving inhibitory control or response selection under conflict

The impact of STN DBS in PD on a number of other tasks that require inhibition of prepotent responses has been examined. The anti-saccade task requires volitional suppression of an innately prepotent response toward a peripherally presented target in order to make a saccade in the opposite direction (Isoda and Hikosaka, 2011). In 32 PD patients with STN DBS tested on medication, Yugeta et al. (2010) found that STN DBS *improved* the ability to suppress unwanted saccades to the cue stimulus in a memory-guided saccade task, but in the anti-saccade task prosaccades were not suppressed. The lack of effect of STN DBS on the anti-saccade task is unexpected and the results with the memory-guided saccades suggests that STN stimulation in PD improves inhibitory control over reflexive saccades, similar to the findings with manual responses on the stop signal task in some studies (van den Wildenberg et al., 2006; Swann et al., 2011; Mirabella et al., 2012).

STN DBS in PD produced some interesting results on the Simon task. In this task, an irrelevant stimulus dimension (e.g., side of the screen stimulus is presented on) can trigger a strong prepotent response that can interfere with selection and execution of the correct response. Wylie et al. (2010) presented blue or green circles to the left or right of a central fixation point to which patients had to respond by pressing right or left buttons with the right or left thumbs, respectively. While the side of the presentation of the stimulus is irrelevant, RTs to stimuli presented in the left visual field for example are faster with the left than with the right hand. The irrelevant stimulus dimension triggers a response with the hand corresponding to the side of stimulus presentation which needs to be inhibited so that the correct response can be selected and executed. The interference with performance due to this response conflict is called the Simon effect. RTs were on average 61 ms faster but accuracy on conflict trials was reduced with STN DBS on than off. By investigating the entire RT distribution, they found that in the fastest part of the RT distribution, STN DBS increased the number of fast premature response captures by the irrelevant stimulus feature, i.e., increased errors relative to DBS off. STN DBS also significantly reduced the magnitude of the Simon effect for the slowest incongruent responses, that is it improved the efficiency of inhibition of the incongruent responses for the slowest part of the RT distribution in the correct trials. These results suggested two temporally dissociable effects of STN stimulation in PD: an early increased automatic response capture by the irrelevant stimulus dimension reflecting impulsivity but a later improved interference control of the slowest responses, which the authors speculated may, respectively, reflect greater responsiveness of the STN with stimulation to inputs from the pre-SMA and IFC. As the STN-induced change in accuracy was limited to the conflict trials and accuracy on non-conflict trials was not altered, it was proposed that this selective effect argues against STN DBS producing a global shift in SATs.

In the latest study, Zavala et al. (2013) recorded LFPs from the STN while PD patients performed an Eriksen flanker task. They reported that correct fast incongruent trials similar to congruent trials had cue-locked STN theta band activity which showed phase alignment across trials followed by a peri-response increase in theta power, suggesting that the distractor flankers were successfully ignored. In contrast, correct incongruent trials with longer RTs had a relative reduction in theta phase alignment followed by higher theta power. It was concluded that STN is involved in processing of congruent and incongruent responses and response selection under conflict and that STN LFPs reflect conflict-related changes.

## Summary and conclusions

The studies which have investigated the effect of STN DBS on tasks requiring inhibition of prepotent responses are summarized in Table 1. As reviewed above, the majority suggest that STN DBS impairs inhibitory control over prepotent responses or in situations of decision conflict. The most inconsistent results are for the stop signal and go no go RT tasks. For the stop signal task, some studies suggest that inhibitory control on this task as measured by the SSRT is improved (van den Wildenberg et al., 2006; Swann et al., 2011; Mirabella et al., 2012), while others found prolongation/worsening of SSRTs (Ray et al., 2009; Obeso et al., 2013) with STN stimulation. As discussed above, there are many methodological factors that could have contributed to these divergent results. For go no go RTs, while no effects of STN DBS were found by van den Wildenberg et al. (2006); Hershey et al. (2004, 2010) and Ballanger et al. (2009) found that STN DBS induced inhibitory deficits reflected in increased commission errors. Furthermore, the work of Hershey and colleagues suggests that such STN DBS induced deficits in action restraint on the go no go task were only present when the response was prepotent (83% target rate) but not when there were fewer go trials (50%) which reduced the prepotency of the response. This group's subsequent work also suggests that stimulation through the ventrally located contacts of the implanted electrodes is associated with reduced discriminability in the go no go task (Hershey et al., 2010). The precise location of the active electrode contact in the STN is an important consideration in determining the effects of STN DBS on executive control. With exceptions (Hershey et al., 2010), in the majority of studies deficits in inhibitory and executive control have been reported for STN stimulation through contacts that are effective in controlling the motor symptoms of PD, suggesting that the active contacts are located in or near the sensorimotor section of the STN. However the influence of contact position in the STN to the observed effects on inhibitory and executive control needs to be directly examined in future studies.

**Table 1:** **Studies which have investigated the effect of deep brain stimulation (DBS) of the subthalamic nucleus (STN) in Parkinson's disease on tasks involving inhibition of prepotent responses, response selection under conflict or decision-making under conflict**.

**Investigators**		**Medication status**	**Worse with STN DBS**	**Unchanged with STN DBS**	**Improved with STN DBS**
2000	Jahanshahi	Off	Stroop interference task		
2002	Schroeder	Off	Stroop interference task		
2004	Hershey	Off	Go no Go RT with high target frequency	Go no Go RTs with lower target frequency	
2006	van den Wildenberg	On		Go no Go RTs	Stop signal RT task
2006	Witt	On	Stroop interference task		
2007	Thobois	Off	Fast-paced RNG		
2007	Frank	On	Probabilistic decision making under high conflict		
2009	Ballanger	Off	Go no go RT		
2009	Ray	On	Stop signal RT task		
2010	Wylie	On	Simon Task—fast responses		Simon task—slow responses
2010	Hershey	Off	Go no Go RT—with ventral STN DBS		
2010	Greenhouse	On		Stop signal RT task—DBS of ventral vs. dorsal contacts	
2010	Yugeta	On		Anti-saccade task	Memory guided saccades
2011	Swann	On			Stop signal RT task
2012	Mirabella	Off			Stop signal RT task
2011	Cavanagh		Probabilisitic decision making under high conflict		
2012	Coulthard	On and Off	Probabilistic decision making requiring integration of conflictual information		
2013	Favre	On			Release of proactive inhibition in unwarned simple RT
2013	Obeso	On	Conditional stop signal RT task		
2013	Green	On	Moving dots task		
in preparation	Pote	On	Moving dots task		

In the context of the majority of the studies summarized in Table 1 demonstrating STN stimulation induced deficits in inhibitory control, it is surprising that STN DBS did not adversely affect the inhibition of pro-saccades in the anti-saccade task although it improved inhibition on the memory guided task, by reducing reflexive saccades to cue presentation. Similarly, negative results were reported by Torta et al. (2012) who examined risk taking behavior and delay aversion, both characteristics of impulsivity, on the Cambridge Gambling task. STN DBS in PD had no effect on delay aversion or risk taking on this task. Using the Iowa Gambling task, which involves decision making under uncertainty, Czernecki et al. (2005) and Oyama et al. (2011) also found that DBS surgery or acute manipulation of stimulation had no overall effect on decision making and risk taking and choice from advantageous vs. non-advantageous decks on this task. The only effect found was that in the Oyama et al. (2011) study performance was worse on the last block of trials with DBS on relative to DBS off. STN DBS-induced worsening of performance on the last block did not correlate with levodopa equivalent dose but was associated with depression scores and DBS through ventral contacts. van Wouwe et al. (2011) reported that STN DBS improved learning of stimulus-action-reward associations on a probabilistic task. From the results of these studies, it is clear that not all forms of impulsivity are detrimentally affected by STN DBS in PD. In light of the multi-faceted nature of impulsivity noted above, future studies need to examine the impact of STN DBS on other components such as the ability to delay gratification and performance on tasks such as the delay discounting task.

Table 2 summarizes the studies that have recorded LFPs or single neuronal activity of the STN during tasks that involve inhibitory control of prepotent responses, most of which were also described above. A range of tasks, go no go RTs, moralistic or probabilistic or economic decision making under conflict, the stop signal RT task, the Stroop, RNG and the Eriksen flanker task were used across these studies. The results of all of these studies concur that activity in the STN itself is modulated in relation to presence of conflict or inhibition of prepotent responses and counteract alternative interpretations such as antidromic stimulation of the cortex or stimulation spread to other structures. Taken together, the results of these electrophysiological studies provide evidence for direct involvement of the STN in inhibitory and executive control and suggest that while theta band activity may reflect evaluative processes and presence of conflict, beta band activity signals preparation and motor readiness, whereas oscillation in the gamma band represents more discrete motor readiness or vigor and gating of action performance/cancellation (Cavanagh and Frank, 2013).

**Table 2:** **Studies recording local field potentials or intraoperative micro-electrode recording of neuronal activity from the subthalamic nucleus (STN) in patients with Parkinson's disease**.

**Investigators**		**Medication status**	**Task**	**Type of recordings**	**Main findings**
2004	Kuhn	Off	Go no go RT	Local field potentials from STN	Increase in beta activity after IS on no go trials relative to go trials.
2011	Fumagalli	On	Moralistic decision-making with or without conflict	Local field potentials from STN	Increased STN activity in low frequency (5–13 Hz) range with conflictual than non-conflictual moralistic decisions.
2011	Cavanagh	?	Probabilistic decision making under high or low conflict	Scalp EEG Intraoperative recordings from STN	Increased theta-band (4–8 Hz) activity over mPFC related to increasing response threshold under high conflict reversed by STN DBS.
2012	Ray	On	Stop Signal Task	Local field potentials from STN	Onset of beta rebound correlated with SSRTs but no differences in beta rebound between successfully inhibited and failed inhibition trials.
2012	Zaghloul	Off	Probabilistic decision making under conflict	Intraoperative microelectrode recordings	Spiking activity in STN increases with degree of decision conflict.
2012	Brittain	On	Stroop Interference Task	Local field potentials from STN	Earlier beta rebound prior to response on correct incongruent trials and after response on incorrent trials.
2013	Anzak	On	Paced RNG	Local field potentials from STN	Increased STN activity in gamma band (45–60 Hz) during RNG relative to control counting task and negatively correlated with habitual counting (count score 1).
2013	Alegre	On and off	Stop signal task	Local Field Potentials from STN	Successful inhibition associated with decrease gamma power and cortico-subthalamic coherence which was absent in the 4 patients with impulse control disorders.
2013	Zavala	On	Eriksen flanker task	Local field potentials from STN	Incongruent trials with fast RTs similar to congruent trials showed cue-locked STN theta band activity with phase alignment across trials and periresponse increase in theta power, which were not observed for incongruent trials with slower RTs.

?, not specified; mPFC, medial prefrontal cortex; SSRTs, stop signal reaction times; RT, reaction time; RNG, random number generation.

In the imaging studies, as STN DBS alters behavior as well as the pattern of brain activation, it is not clear whether this altered pattern is a cause or effect of the altered behavior. However, both are induced by manipulation of STN activity and output. Nevertheless, there is convergent evidence from behavioral and imaging studies of the effect of STN DBS in PD and LFP or intraoperative neuronal recordings from the STN in PD, confirming a role for the STN in inhibitory control over prepotent responses and response selection under conflict during a range of tasks.

## Theoretical implications and future directions

The evidence reviewed supports both of the theoretical frameworks outlined above. The evidence supports the proposal (Frank, 2006; Frank et al., 2007) that alteration of STN activity with STN DBS interferes with the normal function of the STN to increase the response threshold depending on context or situation, to prevent premature and impulsive responses and to allow time for further information accumulation before a decision is made. Support is also provided based on the evidence reviewed above for speed-accuracy trade-off models which attributed a role to the STN in modulating response thresholds and influencing speed-accuracy trade-offs (Bogacz et al., 2010; Mansfield et al., 2011). As recently noted (Jahanshahi, 2013), what remains unclear is whether it is conflict *per se* (Cavanagh et al., 2011; Fumagalli et al., 2011; Zaghloul et al., 2012; Zavala et al., 2013), choice difficulty (Zaghloul et al., 2012; Green et al., 2013), choice accuracy (Zaghloul et al., 2012), the appetitive/aversive valence of the choices (Frank et al., 2007), information integration (Coulthard et al., 2012), adoption of a risk-taking strategy (Rosa et al., 2013) or simply time pressure (Pote et al., in preparation) that influences STN activity and engages it to dynamically modulate response thresholds. These possibilities need to be directly investigated and disentangled in future studies. Furthermore, it is possible that the STN involvement in modulating response thresholds and inhibitory and executive control operates across domains, motor, cognitive and limbic. Such cross-domain generality of the inhibitory role of STN also would be an interesting topic for investigation in future studies. To date, with exceptions (Favre et al., 2013; Obeso et al., 2013), the major focus of the literature on the impact of STN DBS on inhibitory control has been mainly on global reactive inhibition. Proactive and selective inhibition have greater parallels in daily life and, therefore, their investigation is more relevant to understanding the impact of STN DBS on the post-surgical behavior and functioning of PD patients and should be the focus of future study.

Successful performance on the Stroop interference task, fast-paced RNG, the Simon effect task, and the Eriksen flanker task necessitate suppression of habitual and automatic prepotent responses and controlled and strategic selection of alternative responses. The above evidence suggests that STN DBS interferes with this process and thus also supports the proposal that ordinarily the STN implements a switch signal from the frontal cortex to shift from automatic to controlled processing (Isoda and Hikosaka, 2008). In fact, an alternative interpretation of all the evidence showing STN modulated activity with decision or response conflict or task difficulty could be that the STN is signaling to the thalamus and the cortex to “bring more attentional resources” (Whitmer and White, 2012), which would also be consistent with a switch from automatic to controlled processing. The task-specific increase in LFP gamma band activity in the STN in relation to attention-demanding tasks such as paced RNG (Anzak et al., 2013) or verbal fluency (Anzak et al., 2011) may signify such demand for increased attentional resources. An unresolved question is whether STN DBS has detrimental effects only on higher order aspects of executive and inhibitory control or if relatively lower level modulation of response speed is at the heart of the observed deficits. There is some indication from the available evidence that the STN DBS induced deficits in executive and inhibitory control are observed only in conditions of high demand for cognitive control for example on go no go RTs with high but not low target rates (response more prepotent in former case) (Hershey et al., 2004), or when decision-making on win-win but not lose-lose high conflict trials (higher motivational salience in the former case) (Frank et al., 2007). This question needs to be addressed in future studies.

In their influential “paradox of surgery” paper, Marsden and Obeso (1994) posed the question of why disruption of basal ganglia output to the cortex with DBS or lesioning of the STN, GPi or thalamus not impair movement or behavior. They suggested that “Loss of their output to premotor regions might not grossly impair routine movement; the remainder of the distributed system could cope adequately in ordinary circumstances. However, loss of this basal ganglia contribution might impair motor flexibility and adaptation.” The evidence reviewed above and summarized in Table 1, indicates that SN DBS surgery does impair aspects of non-routine behavior in PD and results in a deficit in inhibitory control over prepotent responses and executive control in situations that require response selection under conflict. These deficits resolve the “paradox of surgery.”

## Clinical implications and future directions

A number of case studies have documented that STN DBS induces problems with inhibition of prepotent complex behaviors such as pathological laughter (Krack et al., 2001), pathological crying (Bejjani et al., 1999), pathological gambling (Smeding et al., 2007) or an architects' compulsion to draw female nude figures after surgery (Witt et al., 2006). Hypomania in the immediate post-operative phase has been documented (e.g., Herzog et al., 2003). Some of the other psychiatric complications induced by STN DBS in PD are debated. While STN DBS has been linked with post-surgical de novo emergence of ICDs such as pathological gambling or shopping in some samples (Smeding et al., 2007; Halbig et al., 2009; Lim et al., 2009), others have reported improvement of ICDs with STN DBS in PD (Ardouin et al., 2006; Lim et al., 2009). Similarly, while increased impulsivity and impaired executive control with STN DBS may also contribute to the increased risk of suicides documented in a retrospective study in a minority of cases following STN DBS surgery (Voon et al., 2008), recent prospective evidence has not found any such increased suicide risk in association with STN DBS in PD (Weintraub et al., 2013). Social disintegration, with breakdown of marriage and failure to resume work despite improvement of motor symptoms and function, have been documented following STN DBS in several centers (Houeto et al., 2006; Schüpbach et al., 2006). However, it remains unclear whether the deficits in executive control and heightened impulsivity documented above contribute to these psychiatric and social problems documented following STN DBS surgery in PD as there is no direct evidence available linking the two or whether other more complex psychological or social processes are responsible. To date, only the investigations by Rodriguez-Oroz et al. (2011) and Rosa et al. (2013) relate STN activity to ICDs in PD albeit, in cases who had these problems prior to surgery. It is necessary to directly examine the association between deficits in inhibitory and executive control on experimental tasks and psychiatric and behavioral side-effects of STN DBS in PD in future studies. Furthermore, the clinical significance of the deficits in inhibitory and executive control induced by STN DBS reviewed above for the everyday cognitive functioning of the patients in their daily lives has not been examined to date and remains unknown and is clearly a topic for future investigation.

The improvement of the motor symptoms of PD associated with STN DBS often results in reduction of dopaminergic medication after surgery which can cause apathy, a motivational deficit. Such alteration of motivational state which can in turn change salience or conflict detection, is also relevant to the study of the effect of STN DBS on inhibitory and executive control, and associations of post-surgical apathy with STN stimulation induced deficits in inhibition of prepotent responses and response selection under conflict should be considered.

Furthermore, the key question that arises is whether the deficits in inhibitory and executive control documented above are present in varying degrees in all operated cases. If the answer to this question is “yes,” then a further pertinent question is why are the sequalea of such deficits in executive control not particularly evident in everyday life? Is there some compensatory mechanism operational? If the answer to the first question is “no” and not every operated patient shows deficits in inhibitory and executive control, then a further clinically relevant question is what factors determine which patients develop deficits in inhibitory and executive control. The pre-operative levels of executive functioning, individual differences in executive control (Braver et al., 2010) and predisposition to impulsivity, and the precise location of the implanted electrodes in the STN are likely to be some of the pertinent factors.

STN DBS has been used in treatment of other patients groups, such as those with dystonia (e.g., Kleiner-Fisman et al., 2007) or obsessive compulsive disorder (OCD) (Mallet et al., 2008). In dystonia, reduced cortico-cortical inhibition has been documented (e.g., Edwards et al., 2003). In OCD, obsessions reflect loss of inhibitory control over thoughts that become recurrent and intrusive and distressing and compulsions represent loss of inhibitory control over “safety” behaviors that become repetitive and are engaged in to reduce the anxiety associated with the inflated sense of perceived danger. In OCD both obsessions and compulsions are prepotent and although they are resisted by the patient, compulsions are nevertheless executed. Does STN DBS in dystonia or OCD produce similar or different effects on inhibitory and executive control as in PD? In OCD, it has been proposed that STN DBS may change rigidity to impulsivity/flexibility and that with such increased flexibility the patients are no longer bothered by their obsessions and are no longer driven to execute their compulsions (Krack et al., 2010). Patients with OCD have prolonged SSRTs on the stop signal RT and show deficits on the Stroop task (Chamberlain et al., 2005), indicative of deficits in inhibitory and executive control. Evidence suggests that the STN is also overactive in OCD, but perhaps not to the same extent as in PD (Piallat et al., 2011) and neuronal recordings from the STN revealed associations with doubt and checking behavior during performance of a matching to sample task (Burbaud et al., 2013). It would be interesting to determine how performance on tasks entailing inhibitory and executive control are altered by change of STN overactivity by STN DBS in OCD.

Disruption of the STN activity with DBS could have other as yet unknown implications for the ability of PD patients to exert executive control in making adaptive decisions during conditions of high-conflict in daily life, causing them to revert to automatic/status quo responses even though these may be sub-optimal. Future research would be well placed to examine the potential link between the inhibitory and executive control impairments reviewed here and psychiatric outcomes and everyday cognitive functioning in the course of daily life following STN DBS which nevertheless is highly effective in controlling the motor symptoms of PD.

### Conflict of interest statement

The author declares that the research was conducted in the absence of any commercial or financial relationships that could be construed as a potential conflict of interest.

## Abbreviations

STN: subthalamic nucleus
DBS: Deep brain stimulation
PD: Parkinson's disease
RT: reaction time
SAT: speed accuracy trade-off
RNG: random number generation
CS1: count score 1
SSRT: stop signal reaction time
LFP: local field potential
pre-SMA: pre-supplementary motor area
ACC: anterior cingulate cortex
DLPFC: dorsolateral prefrontal cortex
IFC: inferior frontal cortex
GPi: internal segment of the globus pallidus
ERS: event-related synchronization.
